# Ceftriaxone improves senile neurocognition damages induced by D-galactose in mice

**DOI:** 10.22038/IJBMS.2019.40344.9556

**Published:** 2020-03

**Authors:** Elham Hakimizadeh, Ayat Kaeidi, Zahra Taghipour, Saeed Mehrzadi, Mohammad Allahtavakoli, Ali Shamsizadeh, Gholamreza Bazmandegan, Jalal Hassanshahi, Mohammad Reza Aflatoonian, Iman Fatemi

**Affiliations:** 1 Physiology-Pharmacology Research Center, Research Institute of Basic Medical Sciences, Rafsanjan University of Medical Sciences, Rafsanjan, Iran; 2 Department of Physiology and Pharmacology, School of Medicine, Rafsanjan University of Medical Sciences, Rafsanjan, Iran; 3 Department of Anatomy, Rafsanjan University of Medical Sciences, Rafsanjan, Iran; 4 Razi Drug Research Center, Iran University of Medical Sciences, Tehran, Iran; 5 Research Center for Tropical and Infectious Diseases, Kerman University of Medical Sciences, Kerman, Iran

**Keywords:** Aging, Ceftriaxone, D-galactose, Mice, Oxidative stress

## Abstract

**Objective(s):**

Ceftriaxone (Cef), a beta-lactam antibiotic, is accompanied by antioxidant and anti-inflammatory properties. It has been shown that Cef has beneficial effects on Alzheimer’s disease. In the current investigation, the effect of Cef in a mice model of aging was investigated.

**Materials and Methods:**

Forty male mice were equally aliquoted into four groups as follows: Control (as healthy normal animals), D-galactose (DG) group (treated with 500 mg/kg/day DG for 6 weeks), DG + Cef group (treated with DG plus Cef 200 mg/kg/day for 6 weeks), and Cef group (treated with Cef 200 mg/kg/day for 6 weeks). A battery of behavioral tests was done to evaluate age-related neurocognitive changes. The activities of catalase (CAT), glutathione peroxidase (GPx), and superoxide dismutase (SOD), as well as the level of malondialdehyde (MDA) in the brain, were measured by biochemical methods. Also, to determine the brain damage, histopathological alterations in the hippocampus were measured using hematoxylin and eosin (H&E) staining.

**Results:**

Our results indicate that neurobehavioral dysfunctions of DG can be prevented by co-administration of Cef. We also found that Cef increases the activity of SOD, GPx, and CAT as well as decreasing the level of MDA in the brain of aged mice.

**Conclusion:**

Based on our findings, Cef declines neurocognitive dysfunctions in the DG-induced model of aging, possibly through its antioxidative properties.

## Introduction

Senescence is a slow and progressive biological process associated with many morphological and biochemical alterations in different organs (1). Many age-related alternations in motor and cognitive performance can occur even in the absence of any specific neurodegenerative diseases including Alzheimer’s or Parkinson’s diseases (2). Currently, it is well established that the synthesis of free radicals and free radical-induced damage increase with age (3). According to this hypothesis, free radicals induced intracellular oxidative damage can adversely affect the cellular function and cause cellular death (4). Furthermore, the function of antioxidants such as glutathione peroxidase (GPx), superoxide dismutase (SOD), and catalase (CAT) decline during aging (5). As reported previously, these alternations in antioxidant capacity could cause various behavioral manifestations in senile people such as anxiety, sarcopenia, and cognitive impairments (6). Ceftriaxone (Cef) is a beta-lactam antibiotic that is used for the treatment of infections caused by gram-positive organisms (7). In addition, Cef exerts other pharmacological effects such as anti-inflammatory and antioxidant (8). Previous reports indicated that Cef is a potent scavenger of free radicals and reduces the level of malondialdehyde (MDA). Moreover, it was demonstrated that Cef increases the activity of enzymatic antioxidant defense systems such as SOD, GPx, and CAT (9). In animal models, pre-treatment with Cef can attenuate stroke-related neurohistological and molecular alternations, which are mediated by glutamate transporter-1 (GLT-1) upregulation (10). It is well established that Cef has therapeutic effects in different brain pathological conditions such as spinal muscular atrophy (11), Huntington’s disease (12), and amyotrophic lateral sclerosis (13). Previous reports have indicated that Cef decreases the loss of hippocampal synaptic plasticity and improves memory performance in aquaporin-4 knockout mice (14). Also, Cef can attenuate the degeneration of dopaminergic and motor impairment in a model of Parkinson’s disease (15). 

The current study was designed to study the effect of Cef on an aging model induced by DG in mice using behavioral, histological, and biochemical approaches. 

## Materials and Methods

***Animals***

Male mice (30±2 g, Trimester) were kept in a room with standard conditions in polypropylene cages (23±2 ^°^C, 12 hr: 12 hr light/dark cycle). All experiments were approved by The Animal Ethics Committee of Rafsanjan University of Medical Sciences (permit no. IR.RUMS.REC.1397.041). 

***Drugs***

Cef and DG were obtained from Sigma-Aldrich Company (Germany). Cef was dissolved in distilled water, and mice were treated intraperitoneally (IP) with 200 mg/kg Cef (16). DG was dissolved in a controlled volume of mice drinking water. Two out of four groups of animals received DG at the dose of 500 mg/kg per 10 ml drinking water (3, 6, 17). Daily doses were administered based on daily body weight measurements, and drug solution was freshly prepared for each series of administrations.

***Experimental protocol***

After two weeks of acclimatization, mice were separated into four groups as follows (n=10): 

1- Control group: healthy normal animals without any intervention

2- DG group: received DG (500 mg/kg) orally for six weeks

3- DG + Cef group: received DG (500 mg/kg) orally plus Cef (200 mg/kg) IP for 6 weeks

4- Cef group: received Cef (200 mg/kg) IP for six weeks

The animal body weights were measured once a week. Twenty-four hours after the last Cef administration, animals were subjected to behavioral tests, and all the behavioral tests were followed up at the same time each day.

***Behavioral tests***

*Days 1 and 2. Anxiety-like behaviors*

Day 1. The elevated plus-maze (EPM) test

The EPM test was used to determine the anxiety-like behavior in rodents. The method was mainly similar to our previous studies (18). EPM includes two open arms (50×10 cm) and two closed arms (50×10×40 cm), each animal was placed in the center of the apparatus facing an open arm and observed for 5 min. The percentage of open arm entries (%OAE) and open arm time (%OAT) were reported as the standard indices of anxiety-like behaviors. Any significant reduction in %OAE and/or %OAT indicates an increased level of anxiety. Total arm entries were reported as an index of locomotor activity (6).

Day 2. Corner test and Open-field (OF) test 

In the corner test, each animal was placed in the center of a clean standard home cage, filled with a wooden bed. Animals were evaluated for 30 sec for the numbers of rearing, corner latency, and corner frequency (19). 

Afterward, animals were placed in the center of an OF (50×50×50 cm) and allowed 5 min of free exploration. The perimeter of the box was divided into two zones: central and peripheral. The activity of the mice was digitally recorded and then analyzed using the Ethovision software package (version 7.1, Netherlands). The sequence of behavioral events was recorded as follows: distance moved (cm), velocity (cm/sec), central time (sec), and peripheral time (sec). At the end of each test, the experimental chamber was completely cleaned with diluted ethanol (10%) (20).

*Days 3-6. Memory assessment*

Day 3. Y-maze 

The Y-maze test was used to evaluate the working memory (21). Each animal was placed in the center of the maze and was allowed to freely explore the maze for 8 min. The sequence of arm entries was visually counted by an investigator. A correct alternation was defined as consecutive full entries (excluding the tail) into each of the three arms. The percent of correct alternation was calculated as the number of correct alternations vs the total number of arm visits. After each experiment, the maze was thoroughly cleaned with diluted ethanol (%10) to prevent the adverse effects of olfactory cues.

*Days 4-6, Passive avoidance (PA) test*

Hippocampal-dependent memory deficit in animals was evaluated using the PA test (22). The apparatus had two-compartments (25×25×25 cm), a dark and a light compartment with a steel rod grid floor and Plexiglas walls that were detached by a guillotine door. The PA test was performed in 3 days. On the first day, mice were placed into the apparatus and allowed to pass freely between the chambers around 5 min for adaptation. On the second day, the animals were placed individually in the light compartment for one minute. After the entrance of the mice into the dark compartment, the door was closed, and an electrical shock (0.5 mA, 50 Hz) was delivered for 2 sec through the grid floor. After 20 sec the animals were removed from the dark compartment and transferred to their home cage. On the third day, the process of the second day was repeated, except that no electrical stimulation was given and the time latency to enter the dark compartment was measured as an index of memory performance. The cut-off time for entering the dark compartment was 100 sec for all days.

*Days 7-9. Sensorimotor and motor function*

Day 7. Adhesive removal test 

The adhesive removal test was used to evaluate the sensorimotor function of the forepaw (23). Briefly, a small adhesive label (1×1 cm) was glued to the radial surface of the right forepaw, and the latency for touching and removing the label was recorded during 3 trials. Finally, the obtained values were averaged. 

Day 8. Rotarod

Motor performance and balance skills of animals were evaluated on an accelerating rotating rod (M.T 6800, Iran). The rotarod speed was increased from 10 to 60 rpm. The cut-off time was fixed at 300 sec. Mice were given three trials with a 30 min rest interval. The time taken for each animal to maintain its balance while walking on top of the revolving rod was measured (24).

Day 9. Swimming exhaustion test 

Physical power was assessed by using the swimming exhaustion test (5). Briefly, the mice were dropped individually into a columnar swimming pool (45 cm height and 20 cm radius) filled with fresh water to a depth of 35 cm (34±1 ^°^C, water temperature) so that mice could not use their tails to support themselves. A ring equivalent to 5% of body weight was attached to the tail root of each mouse. The animal exhaustion time was recorded when they failed to rise to the surface of the water for breathing within 7 sec. 

***Molecular tests***

*Tissue preparation*

24 hr after the swimming exhaustion test, animals of each group were sacrificed. Their brains were immediately removed and divided into two hemispheres: one hemisphere was fixed in 10% phosphate-buffered formalin for histological assessment and the other one was homogenized (1/10 w/v) in ice-cold Tris-HCl buffer (100 mM, pH 7.4), centrifuged at 6000 rpm for 20 min, and the supernatant was collected and stored at −80 ^°^C for estimating biochemical assay (25).

***Biochemical assay in brain homogenates***

The lipid peroxidation was evaluated in the brain using a ZellBio MDA kit (Germany; Cat. number: ZB-MDA-96A) according to the manufacture’s protocol. The activity of CAT was evaluated in the brain using a ZellBio assay kit (Germany; Cat. number: ZB-CAT-96A) according to the manufacture’s protocol. The activity of SOD was evaluated in the brain using a ZellBio assay kit (Germany; Cat. number: ZB-SOD-48A) according to the manufacture’s protocol. The activity of GPx was evaluated in the brain using using a ZellBio assay kit (Germany; Cat.Number: ZB-GPX-96A) according to the manufacture’s protocol. All results were presented as the percentage of the control group (26, 27).

***Histological assessment***

For the histological studies, the fixed samples in formalin were dehydrated with a sequence of ethanol solutions, embedded in paraffin, cut into five μm sections, and stained with hematoxylin and eosin (H&E) for light microscopic examinations. The number of intact pyramidal neurons per 1 mm of the hippocampus was quantified at 400 magnifications. The intact pyramidal neurons of the hippocampus were determined by basophilic cytoplasm, large diameter (more than 10 μm), and discrete nucleoli (28, 29). The average value from five sections was used for each animal. 

***Statistical analysis***

GraphPad Prism software (USA) was used for statistical analysis. The differences between the groups were tested using ANOVA followed by Tukey’s *post hoc* test (repeated measure for weight and one-way for other tests). Data are represented as mean±SEM, with a *P*-value of ≤ 0.05 considered statistically significant.

## Results

***The effect of Cef on body weight***

**Figure 1:**
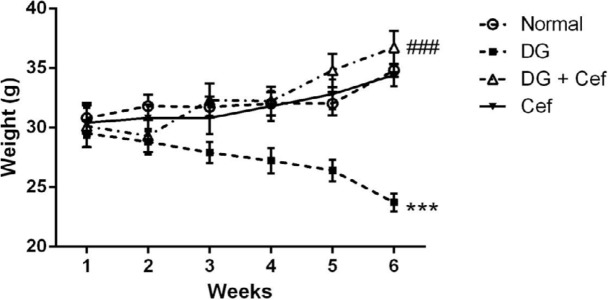
The effect of ceftriaxone (200 mg/kg) on body weight. Values are expressed as mean±SEM. In each group n=10. ***P<0.001 compared with the normal group. ###*P*<0.001 compared with the DG group

DG: D-galactose; Cef: Ceftriaxone

As shown in Figure 1, at the beginning of the experiment, we did not find any significant difference in mean body weight of animals in different experimental groups. DG decreased (*P*<0.001) the bodyweight in comparison with the control group, but DG+ Cef improved the bodyweight compared with the DG group (*P*<0.001). In addition, Cef alone did not significantly affect the bodyweight. 

***The effect of*** ***Cef*** ***on the EPM, corner test, and OF test***

 EPM, corner test, and OF test were used to assess the anxiety-like behavior in different experimental groups. The results of the EPM indicated that DG decreases %OAE (*P*<0.05) and % OAT (*P*<0.001) in comparison with the normal group and administration of Cef along with DG increases %OAE (*P*<0.001) and %OAT (*P*<0.001) in comparison with the DG group. Statistical analysis showed that DG decreases locomotor activity in comparison with the normal group (*P*<0.001), and administration of Cef along with DG increases locomotor activity compared with the DG group. In addition, Cef alone did not significantly affect the indices of EPM (Table 1A). 

In the OF test, It was found that there were no differences in the velocity and time spent in peripheral areas of the field among any groups. Distance index decreased in the DG group in comparison with the normal group (*P*<0.05), and in the DG + Cef group distance index increased in comparison with the DG group (*P*<0.05). DG group showed shorter times of entrance into the central area compared with the control group. But DG + Cef group showed longer times of entrance into the central area compared with the DG group (*P*<0.05). In addition, Cef alone did not significantly affect the indices of the OF test (Table 1B). 

In the corner test, DG group had lower number of rearing (*P*<0.01) and corner frequency (*P*<0.05) than normal animals. Also, the DG group obtained longer times in the variable of corner latency (*P*<0.05). The DG + Cef group revealed a higher number of rearing (*P*<0.05) and corner frequency (*P*<0.01) than the DG group. Moreover, DG + Cef group showed shorter times in the variable of corner latency (*P*<0.001). In addition, Cef alone did not significantly affect the indices of the corner test (Table 1C).

***The effect of*** ***Cef*** ***on Y-maze and PA test***

**Figure 2:**
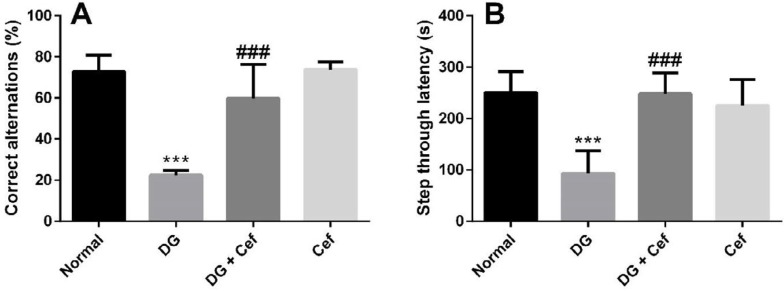
The effect of ceftriaxone (200 mg/kg) on the correct alternations (A), and step-through latency (B). Values are expressed as mean±SEM. In each group n=10. ***P<0.001 compared with the normal group. ###*P*<0.001 compared with the DG group

DG: D-galactose; Cef: Ceftriaxone

We used Y-maze and PA tests to evaluate the working and passive avoidance memory in different experimental groups. In the Y-maze test, the percentage of correct alternations in the DG group significantly reduced compared with normal animals (*P*<0.001). But, in the DG + Cef group, this index significantly increased in comparison with the DG group (*P*<0.001). In addition, Cef alone did not significantly affect the Y-maze test index (Figure 2A).

In the PA test, the index of step-through latency in the DG group was less than that of the normal animals (*P*<0.001). However, treatment of DG receiving mice with Cef increased the step-through latency compared with the DG group (*P*<0.001). In addition, Cef alone did not significantly affect the index of the PA test (Figure 2B)**.**

***The effect of*** ***Cef*** ***on the rotarod, exhaustive swimming test, and adhesive removal test***

**Figure 3:**
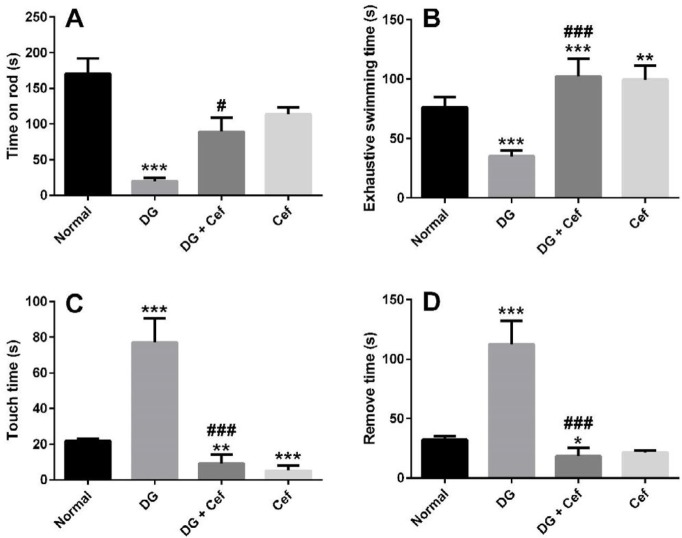
The effect of ceftriaxone (200 mg/kg) on rotarod (A), exhaustive swimming test (B), and adhesive removal test (C and D). Values are expressed as mean±SEM. In each group n=10. **P<0.01 and ***P<0.001 compared with the normal group. #P<0.05 and ###P<0.001 compared with the DG group

DG: D-galactose; Cef: Ceftriaxone

Rotarod, exhaustive swimming test, and adhesive removal test were used to evaluate the physical power and sensorimotor function in different groups. In the rotarod test, the mean of time which animals ran on the rod was shown in Figure 3A. In the DG group, the time which animals ran on the rod significantly decreased in comparison with normal animals (*P*<0.001). But this time significantly increased in the DG + Cef group compared with the DG group (*P*<0.05), which indicated significant functional recovery of locomotor activity. In addition, Cef alone did not significantly affect the rotarod index.

In the exhaustion swimming test, DG group revealed a significant decrease in comparison with the normal group (*P*<0.001) (Figure 3B). Administration of Cef to DG-treated mice significantly increased the exhaustion swimming time compared with DG and normal groups (*P*<0.001). In addition, the Cef group increased this index compared with the normal group (*P*<0.01). 

In the adhesive removal test, DG group, in comparison with the normal animals, latency to touch and remove sticky labels, significantly increased (all *P*<0.001) (Figures 3C and D). In the DG + Cef group, latency to touch and remove sticky labels was significantly decreased in comparison with DG (all *P*<0.001) and normal groups (*P*<0.01 and *P*<0.05, respectively). In addition, Cef group had decreased touch time compared with the normal group (*P*<0.001).

***The effect of Cef on MDA level as well as SOD, GPx, and CAT activity***

**Figure 4.**
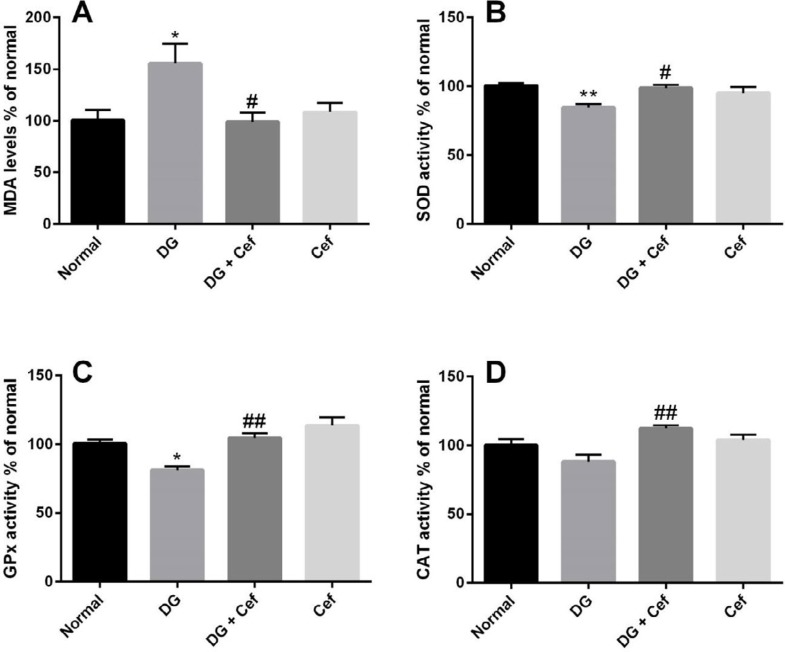
The effect of ceftriaxone (200 mg/kg) on the MDA level (A), SOD activity (B), GPx activity (C), and CAT activity (D). Values are expressed as mean±SEM. In each group n=10. *P<0.05 and **P<0.01 compared with the normal group. #*P*<0.05 and ##*P*<0.01 compared with the DG group

DG: D-galactose; Cef: Ceftriaxone; MDA: Malondialdehyde; SOD: Superoxide dismutase; GPx: Glutathione peroxidase; CAT: Catalase

The MDA level in the DG group was significantly increased in comparison with the normal animals (*P*<0.05) (Figure 4A). In the DG + Cef group, the MDA level decreased in comparison with the DG group (*P*<0.05). In addition, Cef alone did not significantly affect the level of MDA**.**

The SOD activity in the DG group was significantly decreased in comparison with the normal group animals (*P*<0.01) (Figure 4B). In the DG + Cef group, SOD activity increased in comparison with the DG group (*P*<0.05). In addition, Cef alone did not significantly affect the SOD activity.

The GPx activity was significantly decreased in the DG group in comparison with the normal animal (*P*<0.05) (Figure 4C). Administration of Cef significantly increased GPx activity in comparison with the DG group (*P*<0.01). In addition, Cef alone did not significantly affect the GPx activity. 

The CAT activity did not change in the DG group in comparison with the normal animals (*P*<0.05) (Figure 4D). The CAT activity in the DG + Cef group increased in comparison with the DG group (*P*<0.01). In addition, Cef alone did not significantly affect the CAT activity.

***The effect of Cef on histopathological alternations***

**Figure 5:**
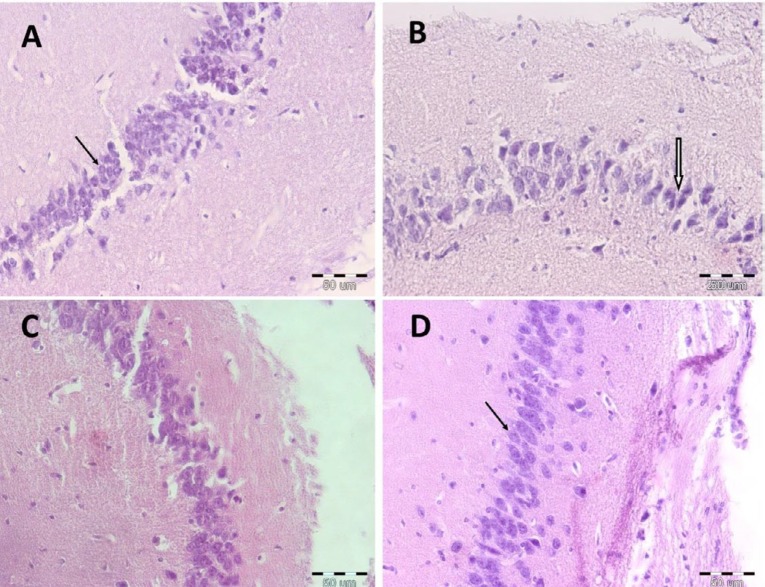
The effect of ceftriaxone (200 mg/kg) on pyramidal neurons of the hippocampus. Normal group (A), DG group (B), Cef + DG group (C), and Cef group (D). Line arrow: normal pyramidal neurons of the hippocampus with euchromatin and clear nucleoli; Arrow: pyramidal neurons of the hippocampus of DG treated mice with pyknotic and dense nucleoli

DG: D-galactose; Cef: Ceftriaxone

In normal and Cef groups, the histopathological morphology of the hippocampus was normal, and the pyramidal neurons have clear nucleoli and cytoplasm (Figure 5). In the DG group, the extent of injury was revealed, and the pyramidal neurons had a shrunken appearance as well as the pyknotic and dense nucleoli. Moreover, administration of Cef to DG-treated mice attenuated these histopathological alternations in the hippocampus neurons.

***The effect of*** ***Cef*** ***on neuron survival of the hippocampus***

**Figure 6.**
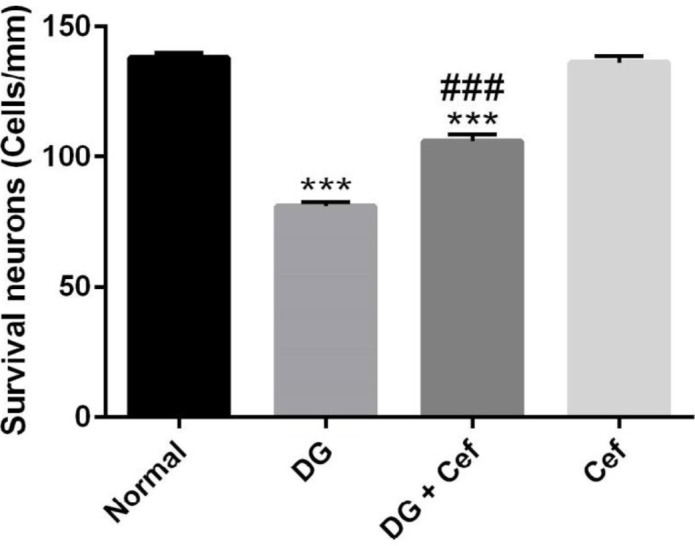
The effect of ceftriaxone (200 mg/kg) on neuron survival of the hippocampus. Values are expressed as mean±SEM. In each group n=10. ***P<0.001 compared with the normal group. ###*P*<0.001 compared with the DG group

DG: D-galactose; Cef: Ceftriaxone

The results of neuron survival of the hippocampus showed that the number of normal neurons in DG treated animals was reduced compared with normal animals (*P*<0.001) (Figure 6). On the other hand, Cef increased the number of normal neurons in DG treated animals (*P*<0.001). In addition, Cef alone did not significantly affect the number of normal neurons. 

## Discussion

The results of the current study clearly implied that administration of DG caused severe aging-related manifestations such as bodyweight reduction, cognitive and sensorimotor impairments, declined physical power, and increased anxiety-like behavior. On the other hand, DG decreased the brain GPx, SOD and CAT activities, as well as increased brain level of MDA. However, Cef could attenuate these deteriorating effects of DG. 

Aging induced by DG serves as a well-established model for performing anti-aging studies (17). A lot of evidence has demonstrated that DG induced accelerating aging via increasing the oxidative stress in different organs (30, 31). It is well-established that DG converts to hydrogen peroxide (H_2_O_2_), which subsequently produces hydroxide ions (OH^-^). Moreover, these types of reactive oxygen species increase the production of MDA, the important biomarker of oxidative damage (32). On the other hand, increased levels of these free radicals reduces the capacity of antioxidants enzymes such as SOD, CAT, and GPx (33). 

Memory impairment is an important feature of aging and age-related neurological disorders (34). Consistent with these studies, our results also showed that Cef (200 mg/kg) improves the performance of passive avoidance and working memory. Previous studies showed the beneficial effects of Cef on cognitive functions in different conditions such as Parkinson’s disease (35, 36), focal cerebral ischemia (37), and Alzheimer’s disease (16). It seems that these effects on learning and memory performance are due to the neuroprotective effects of Cef via decreases in glutamatergic hyperactivity (38). Accordingly, Cef may improve the memory impairments in aging mice through the neuroprotective effects. 

It is well established that the activities of SOD, CAT, and GPx decrease with increasing age (39). Meanwhile, the chain reaction of lipid peroxidation is accelerated (40). The present study showed that administration of DG decreased SOD, CAT, and GPx antioxidant enzyme activity as well as increasing the MDA level. On the other hand, our data indicated that administration of Cef (200 mg/kg) could reverse these changes in DG-treated mice. Amin *et al*. (2014) indicated that on days 3 and 7 after chronic constriction injury (CCI), Cef decreased spinal cord levels of MDA in rats (8). It is well established Cef has a potent antioxidant activity via increasing the level of glutathione (41, 42). In line with our study, Bisht *et al.* (2014) have reported that administration of Cef (100 and 200 mg/kg) significantly decreased MDA levels and increased glutathione and CAT activity in striatum and cortex of parkinsonian rats (43). Also, in another study, it was demonstrated that treatment with Cef significantly increases the GPx and SOD activities as well as decreasing MDA levels in rat brains exposed to ischemia (44). Therefore, Cef will have the potential to be further investigated as an antioxidant medicine in the attenuation of aging-related diseases.

Anxiety is a prevalent problem in late life, elevating health care and social costs (45). DG administration leads to oxidative stress, which is likely to result in anxiety-like behavior (46). In the present study, anxiety-like behavior increased in the DG group in comparison with the normal animals. On the other hand, Cef decreased these behaviors in DG treated animals. On the other hand, Kang *et al.* (2017) showed that Cef reduced anxiety-like behaviors of withdrawn rats via the GLT-1 up-regulation (47). Accordingly, it is possible that Cef decreases anxiety-like behaviors in aging animals via antioxidative effects and the glutamate pathway. 

The locomotor activity can be affected by many factors such as diet, genetics, hypothalamus functions, as well as age (48). It is well established that aging animals have a poor motor function (49). Previous studies demonstrated that DG at high and moderate doses significantly decreased the locomotor activity of the mice (50). Also, researchers demonstrated that DG reduced the skeletal muscle strength via increasing the mitochondrial dysfunction (51). Our results showed that the muscle strength reduced by DG compared with the normal animals. Also, we noticed that administration of Cef (200 mg/kg) significantly improved the physical fatigue in rotarod and forced swimming capacity tests compared with the DG-treated mice. In addition, for the first time, we showed that DG reduces the sensorimotor function by using the sticky test. We found that Cef improved these deleterious effects on sensorimotor function in DG-treated mice. Similar to the results of our study, it has been observed that Cef ameliorates motor deficits in a model of Parkinson’s disease in rats by increasing glutamate transporter expression (15). On the other hand, a substance with antioxidant properties could increase the physical power and locomotor activity in normal and pathological conditions. Moreover, the antioxidant activity of Cef has been confirmed in previous reports. 

In addition, our histopathological observations are in agreement with the findings of behavioral and biochemical tests. Our histopathological results showed the apoptosis of neurons in the hippocampus of DG treated animals. Also, Cef has protective effects on the hippocampus neurons of DG treated animals. It was reported that Cef decreased neuronal apoptosis in the hippocampus and had a tendency to reduce the neuronal loss in the nigrostriatal dopaminergic system in male parkinsonian rats (52). 

In summary, the current study revealed that administration of Cef was effective in mitigating the aging behavioral manifestations induced by DG. Also, Cef reduced the oxidative stress via increasing the activities of SOD, GPx, and CAT as well as decreasing the levels of MDA to protect against DG induced aging. Moreover, Cef restored the survival of hippocampus neurons in the accelerated aging model. Therefore, these results suggest that Cef provides a promising pharmacological approach against age-related conditions.

**Table 1:** The effect of ceftriaxone (200 mg/kg) on the elevated plus-maze, open- field test, and corner test

	**Normal**	**DG**	**DG+Cef**	**Cef**
**A: ** **Elevated plus-maze** ** test**				
Open arm entries (%)	30.65±2.478	14.97±2.922*	40.89±3.986^###^	43.31±1.506
Open arm time (%)	27.79±1.876	9.229±2.602***	34.371±1.878^###^	35.391±2.022
Total arm entries (n)	26±1.19	14.6±0.927***	21.67±1.833^#^	30.80±1.2
**B:** ** Open- field test**				
Distance moved (cm)	9097±843.9	5371±524.6*	8249±609.8^#^	9106±861.6
Velocity (cm/s)	29.61±2.481	19.68±2.568	25.93±2.193	30.35±2.872
Central time (s)	36.56±3.630	19.68±1.218*	23.90±3.762^#^	50.01±5.367
Peripheral time (s)	258.2±5.561	276.3±3.779	266.9±3.755	246.7±6.763
**C: Corner test**				
Corner frequency (n)	6.111±0.454	3.6±0.4*	6.625±0.595^##^	7.4±0.509
Number of rearing (n)	2.125±0.295	0.5±0.223**	2±0.378^#^	2.6±0.4
Corner latency (s)	23.29±0.42	30.20±1.281*	17±2.352 ^###^	19.80±0.663

Values are expressed as mean±SEM. In each group n=10. *P<0.05, **P<0.01, and ***P<0.001 compared with the normal group, # *P*<0.05, ## *P*<0.01, and ### *P*<0.001 compared with the DG group. DG: D-galactose; Cef: Ceftriaxone
